# Supplementary material for: Maternal occupational exposures during early stages of pregnancy and adverse birth outcomes in the NINFEA birth-cohort
Source: PLoS One. 2025 Jan 14;20(1):e0313085. doi: 10.1371/journal.pone.0313085 (PMC11731713; doi:10.1371/journal.pone.0313085)
Supplement: S1 File — (DOCX) [file pone.0313085.s001.docx]

**Supplementary Materials**

**Supporting Table 1.** Questions and all available exposures from the questionnaire to collect information on women working conditions during the first trimester of pregnancy. The transformed exposures from categorical to binary were used for the analysis.

| **Variable (item)** | **Question** | **Original exposures** | **Binary exposures** |
| --- | --- | --- | --- |
|  | How much energy expenditure was required for your work during pregnancy? | - Few - Sufficient - High | - Few + Sufficient - High |
|  | How was your working activity during the previous year during pregnancy? | - Repetitive/Tedious - Demanding/Onerous - Both - None of the two | *This exposure has been divided into two separate exposures:*  1.1   - Repetitive/Tedious + Both - None of the two   1.2   - Demanding/Onerous + Both - None of the two |
|  | Did you work the night shifts during the year during pregnancy? | - Yes - No |  |
|  | How would you define your workplace? | - Very noisy - Noisy - Little noisy - Not noisy | - Very noisy + noisy - Little noisy + not noisy |
|  | Did you work in an indoor workplace where other people were smoking during the first trimester of pregnancy? | - Yes - No |  |
|  | What do you think about your daily working activity during the first trimester of pregnancy? | - Extremely stressed - Very stressed - Moderately stressed - Little stressed - No stressed | - Very stressed + extremely stressed - Moderately stressed + little stressed + moderately stressed |
|  | Have you been exposed to chemicals or fumes during the first trimester of pregnancy? | If yes:   - Lead - Chromium - Diesel Engine Exhaust - Pesticides - Varnishes - Solvents - Inks - Industrial oils - Formalin/Formaldehyde - Chemotherapeutics - Anaesthetic gases - Other chemicals or fumes (reported in free text) - *Dust |  |

* Dust exposure was included in the table list for this study because several women listed it under the category ‘other chemicals or fumes (reported in free text)’.

**Supporting Table 2.** Number of nulliparous working mothers of the NINFEA cohort and corresponding CNO-94 code and CNO-94 job group.

| **CNO-94 codes** | **CNO-94 job group** | **Number of workers** |
| --- | --- | --- |
| 0 | Armed Forces | 2 |
|  | | |
| 1 | Legislators, Senior official and managers | 52 |
| 12 | General managers | 4 |
| 112 | Production and operations department managers | 6 |
| 113 | Other department managers | 4 |
| 1012 | Legislators | 1 |
| 1020 | Senior government officials | 1 |
| 1122 | Production and operations managers in agriculture, hunting, forestry, and fishing | 5 |
| 1124 | Production and operations managers in wholesale and retail trade | 5 |
| 1125 | Production and operations managers in restaurant and hotels | 3 |
| 1126 | Production and operations managers in transport, storage, and communications | 2 |
| 1131 | Finance and administration department managers | 28 |
| 1132 | Personnel and industrial relations department managers | 7 |
| 1133 | Sales and marketing department managers | 16 |
| 1134 | Advertising and public relations department managers | 4 |
| 1136 | Computing services department managers | 3 |
| 1137 | Research and development department managers | 2 |
| 1138 | Other department managers not elsewhere classified | 3 |
| 1220 | General managers in wholesale and retail trade | 22 |
| 1409 | General managers not elsewhere classified | 1 |
|  | ***Total number of subjects working in business and public administration*** | ***188*** |
|  | | |
| 2 | Professionals | 134 |
| 22 | Teaching professionals | 115 |
| 23 | Legal professionals | 2 |
| 24 | Business professionals | 1 |
| 25 | Writers and creative or performing artists | 1 |
| 28 | Teaching associate professionals | 2 |
| 205 | Architects, engineers, and related professionals | 15 |
| 222 | Secondary education teaching professionals | 10 |
| 223 | Other teaching professionals | 58 |
| 239 | Legal professionals not elsewhere classified | 5 |
| 241 | Business professionals in company organisation and administration | 1 |
| 251 | Authors, journalists, and other writers | 2 |
| 261 | Physicists, chemists, and related professionals | 1 |
| 263 | Computer associate professionals | 5 |
| 264 | Technical architect | 1 |
| 265 | Technical engineer | 2 |
| 283 | Other teaching professionals | 2 |
| 291 | Finance and sales associate professionals | 1 |
| 2011 | Physicists and astronomers | 1 |
| 2022 | Statisticians | 6 |
| 2031 | Computer system designers and analysts | 11 |
| 2040 | Architects, town, and traffic planners | 76 |
| 2051 | Civil engineers | 14 |
| 2054 | Mechanical engineers | 2 |
| 2111 | Biologists, botanists, zoologists, and related professionals | 25 |
| 2113 | Agronomists and related professionals | 2 |
| 2121 | Medical doctors | 116 |
| 2122 | Dentists | 7 |
| 2130 | Veterinarians | 26 |
| 2140 | Pharmacists | 43 |
| 2210 | College, university, and higher education teaching professionals | 11 |
| 2220 | Secondary education teaching professionals | 25 |
| 2231 | Education methods specialists | 3 |
| 2239 | Other teaching professionals not elsewhere classified | 3 |
| 2311 | Lawyers | 56 |
| 2312 | Prosecutors | 23 |
| 2320 | Judges | 2 |
| 2411 | Accountants | 19 |
| 2412 | Professionals in human resources | 14 |
| 2413 | Professionals in advertising and public relation | 14 |
| 2419 | Business professional not elsewhere classified | 39 |
| 2431 | Sociologists, anthropologists, and related professionals | 2 |
| 2433 | Philosophers, historians, and political scientists | 20 |
| 2434 | Psychologists | 81 |
| 2511 | Authors, journalists, and other writers | 39 |
| 2512 | Sculptors, painters, and related artists | 9 |
| 2513 | Composers, musicians, and singers | 1 |
| 2515 | Film, stage, and related actors and directors | 4 |
| 2521 | Archivists and curators | 4 |
| 2522 | Librarians and related information professionals | 5 |
| 2613 | Chemists | 7 |
| 2614 | Geologists and geophysicists | 7 |
| 2631 | Analysts of applications and computer programmers of medium level | 33 |
| 2639 | Other computer professionals of medium level | 1 |
| 2640 | Architects, engineers, and related professionals not elsewhere classified | 3 |
| 2711 | Biologists, botanists, zoologists, and related professionals | 1 |
| 2720 | Nursing associate professionals | 132 |
| 2811 | Primary education teaching professionals | 30 |
| 2812 | Pre-primary education teaching professionals | 47 |
| 2911 | Administrative associate professionals not elsewhere classified | 2 |
| 2912 | Social work associate professionals | 2 |
|  | ***Total number of subjects working as scientific and technicians, and professionals*** | ***1322*** |
|  | | |
| 3 | Technician and associate professionals | 13 |
| 30 | Physical and engineering science technicians | 15 |
| 33 | Finance and sales associate professionals | 1 |
| 302 | Physical and chemical engineering technicians | 1 |
| 303 | Computer associate professionals | 7 |
| 307 | Safety and quality inspectors | 1 |
| 313 | Modern health associate professionals (expect nursing) | 3 |
| 331 | Finance and sales associate professionals | 2 |
| 341 | Administrative secretaries and related associate professionals | 4 |
| 353 | Social work associate professionals | 3 |
| 354 | Artistic, entertainment, and sports associate professionals | 5 |
| 3010 | Draughtspersons | 10 |
| 3022 | Civil engineering technicians | 15 |
| 3023 | Electrical engineering technicians | 2 |
| 3026 | Chemical engineering technicians | 5 |
| 3027 | Mining and metallurgical technicians | 1 |
| 3029 | Physical and engineering science technicians not elsewhere classified | 2 |
| 3031 | Computer equipment operators | 6 |
| 3041 | Photographers and image and sound recording equipment operators | 5 |
| 3043 | Medical equipment operators | 12 |
| 3061 | Air traffic controllers | 2 |
| 3072 | Safety, health, and quality inspectors | 1 |
| 3073 | Quality control technician | 5 |
| 3112 | Agronomy and forestry technicians | 2 |
| 3121 | Life science technicians | 28 |
| 3122 | Veterinary assistant | 1 |
| 3123 | Sanitarians | 4 |
| 3124 | Dental assistants | 7 |
| 3129 | Modern health associate professionals | 25 |
| 3131 | Dieticians and nutritionists | 12 |
| 3132 | Optometrists and opticians | 3 |
| 3133 | Physiotherapists and related associate professionals | 31 |
| 3134 | Occupational therapists | 2 |
| 3135 | Speech therapists | 18 |
| 3211 | Primary education teaching associate professionals | 2 |
| 3212 | Special education teaching associate professionals | 3 |
| 3311 | Securities and finance dealers and brokers | 3 |
| 3312 | Insurance representatives | 6 |
| 3313 | Estate agents | 3 |
| 3314 | Travel consultants and organisers | 14 |
| 3316 | Buyers | 3 |
| 3320 | Technical and commercial sales representatives | 29 |
| 3411 | Secretaries | 66 |
| 3412 | Insurance representatives | 1 |
| 3422 | Government tax and excise officials | 1 |
| 3423 | Government social benefits officials | 1 |
| 3531 | Social work associate professionals | 68 |
| 3539 | Equality promoters for women opportunities and support to social promotion | 27 |
| 3541 | Decorators and commercial designers | 5 |
| 3543 | Street, night-club and related musicians, singers, and dancers | 1 |
| 3545 | Athletes, sportspersons, and related associate professionals | 10 |
|  | ***Total number of subjects working as support technicians and professionals*** | ***492*** |
|  | | |
| 4 | Clerks | 1403 |
| 452 | Travel agency and related clerks | 1 |
| 4011 | Accounting and bookkeeping clerks | 23 |
| 4012 | Statistical and finance clerks | 44 |
| 4023 | Transport clerks | 2 |
| 4102 | Mail carriers and sorting clerks | 4 |
| 4510 | Client information clerks | 7 |
| 4521 | Travel agency and related clerks | 2 |
| 4522 | Receptionists and information clerks | 6 |
| 4523 | Telephone switchboard operators | 17 |
| 4601 | Cashiers and ticket clerks | 15 |
| 4602 | Tellers and other counter clerks | 3 |
| 4605 | Debt-collectors and related workers | 1 |
|  | ***Total number of subjects working as administrative-type employees*** | ***1528*** |
|  | | |
| 5 | Service workers and shop and market sales workers | 1 |
| 50 | Housekeeping and restaurant services workers | 4 |
| 529 | Protective services workers not elsewhere classified | 1 |
| 5010 | Cooks | 14 |
| 5020 | Waiters, waitresses, and bartenders | 48 |
| 5030 | Chief cook, waiters, and related workers | 2 |
| 5111 | Institution-based personal care workers | 30 |
| 5113 | Home-based personal care workers | 15 |
| 5121 | Child-care workers | 3 |
| 5130 | Hairdressers, barbers, beauticians, and related workers | 27 |
| 5141 | Travel attendants and travel stewards | 3 |
| 5142 | Car auditors and travel collectors | 1 |
| 5143 | Travel guides | 8 |
| 5221 | National police officers | 9 |
| 5223 | Local police officers | 10 |
| 5230 | Fire-fighters | 2 |
| 5299 | Protective services workers not elsewhere classified | 1 |
| 5310 | Fashion and other models | 1 |
| 5320 | Shop salespersons and demonstrators | 32 |
| 5330 | Stall and market salespersons | 97 |
|  | ***Total number of subjects working as service workers and shop and market sales workers*** | ***309*** |
|  | | |
| 60 | Market gardeners and crop growers | 2 |
| 6011 | Field crop and vegetable growers for agricultural activity in own company | 1 |
| 6022 | Field crop and vegetable growers and employed in gardens and orchards | 1 |
| 6111 | Mixed-animal producers | 2 |
|  | ***Total number of subjects working as skilled agricultural and fishery workers*** | ***6*** |
|  | | |
| 7 | Craft and related trades workers | 31 |
| 70 | Extraction and building trades workers | 1 |
| 73 | Metal, machinery, and related trades workers | 5 |
| 76 | Electrical and electronic equipment mechanics and fitters | 1 |
| 78 | Food processing and related trades workers | 1 |
| 79 | Wood treaters and workers in textile and leather industry | 1 |
| 752 | Blacksmiths, toolmakers, and related trades workers | 1 |
| 773 | Potters, glassmakers, and related trades workers | 1 |
| 780 | Food processing and related trades workers | 1 |
| 793 | Textile, garment, and related trades workers | 2 |
| 794 | Pelt, leather, and shoemaking trades workers | 7 |
| 7030 | Painters and related workers | 1 |
| 7294 | Glaziers | 2 |
| 7611 | Motor vehicle mechanics and fitters | 1 |
| 7711 | Precision-instrument makers and repairers | 12 |
| 7731 | Abrasive wheel formers, potters, and related workers | 1 |
| 7933 | Tailors, dressmakers, and hatters | 2 |
| 7936 | Sewers, embroiderers, and related workers | 2 |
|  | ***Total number of subjects working as craft and related trades workers*** | ***73*** |
|  | | |
| 8 | Plant and machine operators and assemblers | 2 |
| 83 | Drivers and mobile-plant operators | 1 |
| 86 | Motor-vehicle drivers | 1 |
| 812 | Metal-processing plant operators | 1 |
| 815 | Chemical-processing plant operators | 2 |
| 8610 | Taxi drivers of car and vans | 1 |
|  | ***Total number of subjects working as plant and machine operators and assemblers*** | ***8*** |
|  | | |
| 93 | Messengers, porters, doorkeepers, and related workers | 1 |
| 9001 | Street vendors and related workers | 1 |
| 9220 | Doorkeepers, watchpersons, and related workers | 3 |
| 9340 | Vending-machine money collectors, meter readers and related workers | 1 |
| 9351 | Garbage collectors | 2 |
| 9410 | Farmhand and labourers | 2 |
|  | ***Total number of subjects in elementary occupations*** | ***10*** |
|  | | |
|  | ***Total number of working mothers*** | ***3938*** |

**Supporting Table 3.** Sensitivity analysis of maternal occupational exposure according to the JEMs, considering only women with jobs defined with complete information at four digits of CNO-94 (n = 1986).

| **Occupational exposure** | **Birthweight**  **B (95% CI)** | **Preterm birth**  **OR (95% CI)** | **Caesarean section**  **OR (95% CI)** |
| --- | --- | --- | --- |
| **Heat** | -107.4 (-256.9 to 42.0) | 0.74 (0.12 to 2.50) | 1.05 (0.49 to 2.09) |
| **Workload** | 55.8 (-100.8 to 212.4) | / | 1.63 (0.80 to 3.21) |
| **Standing work** | -82.0 (-239.7 to 75.7) | 2.42 (0.70 to 6.39) | 0.53 (0.18 to 1.29) |
| **VDT** | 8.6 (-53.6 to 70.9) | 1.46 (0.89 to 2.32) | 1.22 (0.92 to 1.63) |
| **Sedentary** | -1.5 (-68.4 to 65.3) | 1.54 (0.91 to 2.51) | 1.26 (0.93 to 1.71) |
| **Repetitive movements** | -52.9 (-176.3 to 70.4) | 1.60 (0.54 to 3.79) | 0.51 (0.23 to 1.02) |
| **Detergents** | -19.8 (-88.0 to 48.4) | 0.62 (0.28 to 1.21) | 1.22 (0.88 to 1.68) |
| **Animal dust** | 61.4 (-115.1 to 237.9) | / | 1.04 (0.44 to 2.25) |
| **Endocrine disruptors** | -76.0 (-164.7 to 12.7) | 1.55 (0.73 to 2.97) | 1.27 (0.82 to 1.92) |

**Abbreviations: UV = Ultraviolet; VDT = Video display terminal**

**Supporting Table 4.** Sensitivity analysis of maternal occupational exposure according to the JEMs performed to evaluate differences in exposure assignment using imputation from the JEMs at a 50% threshold for the prevalence of exposure (rather than the 75% threshold used in the main analysis).

| **Occupational exposure** | **Birthweight**  **B (95% CI)** | **Preterm birth**  **OR (95% CI)** | **Caesarean section**  **OR (95% CI)** |
| --- | --- | --- | --- |
| **Heat** | -118.5 (-261.6 to 24.5) | 1.05 (0.25 to 2.93) | 0.75 (0.35 to 1.49) |
| **Workload** | -4.1 (-71.9 to 63.6) | 0.64 (0.30 to 1.20) | 0.85 (0.60 to 1.17) |
| **Standing work** | -10.8 (-59.0 to 37.4) | 0.93 (0.60 to 1.39) | 0.94 (0.74 to 1.18) |
| **VDT** | -6.4 (-48.6 to 35.7) | 0.94 (0.65 to 1.34) | 0.95 (0.78 to 1.15) |
| **Sedentary** | -24.7 (-79.0 to 29.4) | 1.10 (0.70 to 1.67) | 1.03 (0.80 to 1.32) |
| **Repetitive movements** | -43.8 (-99.7 to 11.2) | 1.65 (1.09 to 2.44) | 1.13 (0.87 to 1.45) |
| **Detergents** | 14.3 (-48.5 to 77.2) | 0.75 (0.39 to 1.31) | 0.97 (0.71 to 1.30) |
| **Animal dust** | 114.2 (-56.0 to 284.4) | 0.43 (0.02 to 2.02) | 0.93 (0.40 to 1.96) |
| **Endocrine disruptors** | -61.7 (-148.2 to 24.8) | 1.38 (0.66 to 2.56) | 1.23 (0.82 to 1.82) |

**Abbreviations: UV = Ultraviolet; VDT = Video display terminal**
